# Coccidioidomycosis-associated Deaths, United States, 1990–2008

**DOI:** 10.3201/eid1811.120752

**Published:** 2012-11

**Authors:** Jennifer Y. Huang, Benjamin Bristow, Shira Shafir, Frank Sorvillo

**Affiliations:** Keck School of Medicine of University of Southern California, Los Angeles, California, USA (J.Y. Huang);; and University of California Los Angeles Fielding School of Public Health, Los Angeles (B. Bristow, S. Shafir, F. Sorvillo)

**Keywords:** coccidioidomycosis, mortality, United States, fungi, deaths, Coccidioides immitis, Coccidioides posadasii, Coccidioides spp

## Abstract

Coccidioidomycosis is a fungal disease that occurs throughout the Americas. It is contracted by inhaling spores, which are carried in dust. Therefore, it occurs most commonly in dry areas and in persons who work in dusty conditions (such as agricultural workers, construction workers, military personnel, and archeological site workers). A substantial number of people die of this disease each year, so researchers examined what other factors increase the risk for death. They found that risk for death was highest among men, elderly persons (>65 years), Hispanics, Native Americans, residents of California and Arizona, and those who also had HIV or other immune-suppressive conditions. Physicians should be aware of which patients are at increased risk and should ask patients about their travel history or occupation to determine possible sources of exposure.

## Medscape CME Activity

Medscape, LLC is pleased to provide online continuing medical education (CME) for this journal article, allowing clinicians the opportunity to earn CME credit.

This activity has been planned and implemented in accordance with the Essential Areas and policies of the Accreditation Council for Continuing Medical Education through the joint sponsorship of Medscape, LLC and Emerging Infectious Diseases. Medscape, LLC is accredited by the ACCME to provide continuing medical education for physicians.

Medscape, LLC designates this Journal-based CME activity for a maximum of 1 *AMA PRA Category 1 Credit(s)*^TM^. Physicians should claim only the credit commensurate with the extent of their participation in the activity.

All other clinicians completing this activity will be issued a certificate of participation. To participate in this journal CME activity: (1) review the learning objectives and author disclosures; (2) study the education content; (3) take the post-test with a 70% minimum passing score and complete the evaluation at www.medscape.org/journal/eid; (4) view/print certificate.

**Release date: October 19, 2012; Expiration date: October 19, 2013**

## Learning Objectives

Upon completion of this activity, participants will be able to:

- Assess the epidemiology of coccidioidomycosis
- Evaluate the transmission and clinical course of coccidioidomycosis
- Analyze demographic risk factors for mortality in cases of coccidioidomycosis
- Distinguish comorbid conditions associated with a higher risk of mortality in cases of coccidioidomycosis

## CME Editor

**P. Lynne Stockton, VMD, MS, ELS(D),** Technical Writer/Editor, *Emerging Infectious Diseases. Disclosure: P. Lynne Stockton, VMD, MS, ELS(D), has disclosed no relevant financial relationships.*

## CME Author

**Charles P. Vega, MD,** Health Sciences Clinical Professor; Residency Director, Department of Family Medicine, University of California, Irvine. *Disclosure: Charles P. Vega, MD, has disclosed no relevant financial relationships.*

## Authors

*Disclosures:* ***Jennifer Y. Huang, MPH; Benjamin Bristow, MD, MPH; Shira Shafir, PhD, MPH;*** *and* ***Frank Sorvillo, PhD,*** *have disclosed no relevant financial relationships.*
