# Supplementary material for: Coccidioidomycosis-associated Deaths, United States, 1990–2008
Source: Emerg Infect Dis. 2012 Nov;18(11):1723–8. doi: 10.3201/eid1811.120752 (PMC3559166; doi:10.3201/eid1811.120752)
Supplement: Related texts

# Research article: Coccidioidomycosis-associated Deaths, United States, 1990–2008

**DOI:** 10.3201/eid1811.120752

**Authors:** Jennifer Y. Huang, Benjamin Bristow, Shira Shafir, Frank Sorvillo

**Affiliations:** Keck School of Medicine of University of Southern California, Los Angeles, California, USA (J.Y. Huang);; and University of California Los Angeles Fielding School of Public Health, Los Angeles (B. Bristow, S. Shafir, F. Sorvillo)

## Abstract

Coccidioidomycosis is endemic to the Americas; however, data on deaths caused by this disease are limited. To determine the rate of coccidioidomycosis-associated deaths in the United States, we examined multiple cause–coded death records for 1990–2008 for demographics, secular trends, and geographic distribution. Deaths were identified by International Classification of Diseases, 9th and 10th Revision, codes, and mortality rates were calculated. Associations of deaths among persons with selected concurrent conditions were examined and compared with deaths among a control group who did not have coccidioidomycosis. During the 18-year period, 3,089 coccidioidomycosis-associated deaths occurred among US residents. The overall age-adjusted mortality rate was 0.59 per 1 million person-years; 55,264 potential life-years were lost. Those at highest risk for death were men, persons >65 years, Hispanics, Native Americans, and residents of California or Arizona. Common concurrent conditions were HIV and other immunosuppressive conditions. The number of deaths from coccidioidomycosis might be greater than currently appreciated.

Coccidioidomycosis is a reemerging infectious disease caused by inhalation of airborne spores of the soil fungus *Coccidioides immitis* or *C. posadasii* (*1*). *Coccidioides* spp. are native to arid and desert areas in North America (California, Arizona, Texas, Utah, Nevada, New Mexico, and northern parts of Mexico), Central America, and South America (*2*). The manifestations of infection with either organism are assumed to be identical. *Coccidioides* spp. are found in lower elevation areas that receive <20 inches of rain per year and have warm, sandy soil (*3*). They are usually found 4–12 inches below the surface. Organism growth is enhanced in areas of animal droppings, burial sites, and animal burrows (*4*). Among persons living in coccidioidomycosis-endemic areas, ≈10%–50% have been exposed to *Coccidioides* spp. Each year in the United States, an estimated 150,000 new cases of coccidioidomycosis occur (*5*). The trend of incidence varies by state because of differences in epidemiology, reporting standards, and case definitions. For 2010, the 2 states most affected by coccidioidomycosis, Arizona and California, reported incidence of 186.0 and 11.5 cases per 100,000 population, respectively (*6*–*8*).

The epidemiology of coccidioidomycosis varies by area; environmental factors play a major role (*9*). Exposure to *Coccidioides* spp. varies by season, geographic location (*3*), and condition of the air. Exposure to spores is more common in dusty conditions, e.g., after earthquakes, dust storms, droughts, and other natural disasters that increase the amount of dust in the air (*10*). Persons in certain occupations are at higher risk for exposure to spores, e.g., archeologists (*11*), military personnel, construction workers (*12*), and farmers. Prisons in coccidioidomycosis-endemic areas might place inmates at risk for exposure (*13*).

Pulmonary infection can result from inhalation of 1 spore; however, high numbers of spores are more likely to result in symptomatic disease. With rare exception, animal-to-person or person-to-person transmission does not occur. The incubation period is 1–4 weeks. Most patients are asymptomatic, but others might have an acute or chronic disease that initially resembles a protracted respiratory or pneumonia-like febrile illness primarily involving the bronchopulmonary system. Dissemination to multiple organ systems can occur. Illness is typically characterized by >1 of the following: influenza-like signs and symptoms; pulmonary lesion diagnosed by chest radiograph; erythema nodosum or erythema multiforme rash; meningitis; or involvement of bones, joints, skin, viscera, and lymph nodes (*14*,*15*). Extrapulmonary manifestations occur in 0.6% of the general population, most commonly secondary to hematogenous spread; meningitis carries an especially grave prognosis (*16*). The risk for disseminated disease is significantly higher among men (*17*), those with compromised or suppressed immune systems (e.g., persons with HIV), those receiving corticosteroids, and pregnant women. Risk for disseminated disease also seems to be higher for African Americans and Filipino Americans (*18*).

Despite the potential for coccidioidomycosis to be severe and fatal, studies of deaths associated with coccidioidomycosis in the United States are limited (*19*,*20*). To determine possible risk factors for coccidioidomycosis-associated death, we used US multiple-cause-of-death data to assess demographics, secular trends, geographic distribution, and concurrent conditions.

## Methods

### Data Sources

We analyzed de-identified, publicly available multiple-cause-of-death data from US death certificates from the National Center for Health Statistics for 1990–2008 (*21*,*22*). These death records contained demographic information for each decedent (including age, sex, and race/ethnicity) and geographic information (state of residence and place of death). In addition to designating underlying causes, the physician or coroner can list conditions that are believed to have contributed to the death. These conditions were coded according to the International Classification of Diseases, 9th Revision (ICD-9), for 1990–1998 and International Classification of Diseases, 10th Revision (ICD-10), for 1999–2008 (*23*,*24*). A coccidioidomycosis-associated death was defined as death of a US resident with an ICD-9 code of 114.0–114.9 or an ICD-10 code B38.0–B38.9 listed as an underlying or contributing cause on the death record (*21*,*25*).

### Mortality Rates and Trends

We calculated mortality rates and 95% CIs by using bridged-race population estimates derived from US census data, and we subsequently age-adjusted these rates with weights from the 2000 US standard population data. Mortality rates and rate ratios were calculated by a decedent’s race/ethnicity (white, black, Hispanic, Asian, Native American), year of death, and state of residence by using aggregated data from all years of study to ensure stable rates. Years of potential life lost were calculated by subtracting age at death from 75 for all who died before 75 years of age (*26*). This method was used for consistency with the Centers for Disease Control and Prevention Web-based Injury and Statistics Query and Reporting System (*27*).

### Analysis of Concurrent Illnesses

To identify possible risk factors for coccidioidomycosis-associated death, concurrent conditions that were noted on the death records were compared with those noted on the records of a control group whose deaths were not associated with coccidioidomycosis. Five control decedents were randomly selected and matched to each coccidioidomycosis decedent by 5-year age group, sex, and race. Matched odds ratios and 95% CIs were computed. Concurrent conditions were chosen on the basis of biological plausibility and known or suspected risk factors for death from coccidioidomycosis. Concurrent conditions were identified on the death record as either an underlying or contributing cause (*21*,*25*). All analyses were conducted by using SAS 9.2 software (SAS Institute, Inc, Cary, NC, USA).

## Results

### Demographics

During 1990–2008, a total of 3,089 coccidioidomycosis-associated deaths among US residents were identified; these deaths represent 55,264 years of potential life lost. The overall crude mortality rate was 0.58 per 1 million person-years (95% CI 0.56–0.61); after age adjustment, the mortality rate was 0.59 deaths per 1 million person-years (95% CI 0.57–0.61).

Age-adjusted mortality rates, by year, are shown in Figure 1. A total of 2,202 (>70%) decedents were men, and 887 (28.7%) were women; age-adjusted mortality rates were 0.94 per 1 million person-years and 0.32 per 1 million person-years, respectively. Death associated with coccidioidomycosis was 2.04× (95% CI 2.84–3.26) more likely for men than for women.

**Figure 1:** Age-adjusted mortality rates of coccidioidomycosis, per 1 million persons in the United States, by year, 1990–2008.

Mortality rates were higher for decedents >65 years of age than for those in other age groups (Table 1). Most (603 [19.5%]) deaths occurred at 65–74 years of age; age-specific mortality rate was 1.70 per 1 million person-years. Although the 206 decedents >85 years of age represented only 6.7% of the total deaths, the mortality rate was highest for this group (2.56 deaths/1 million person-years).

**Table 1:** Demographic characteristics, mortality rates, and rate ratios for coccidioidomycosis-associated deaths, United States, 1990–2008*

Characteristic	No. (%)	Age-adjusted mortality rate (95% CI)†	Age-adjusted rate ratio (95% CI)
Overall	3,089 (100)	0.59 (0.57–0.61)	NA
Sex			
F	887 (28.7)	0.31 (0.29–0.33)	Referent
M	2,202 (71.3)	0.93 (0.90–0.98)	3.04 (2.84–3.26)
Race/ethnicity			
White	1,693 (54.8)	0.40 (0.38–0.42)	Referent
Hispanic	747 (24.2)	1.77 (1.63–1.90)	4.38 (4.17–4.60)
Black	392 (12.7)	0.69 (0.62–0.75)	1.70 (1.61–1.80)
Asian	178 (5.8)	1.14 (0.96–1.32)	2.82 (2.69–2.97)
Native American	79 (2.6)	2.56 (1.96–3.15)	6.34 (6.04–6.65)
Age, y			
0–4	8 (0.3)	0.11 (0.03–0.18)	NA
5–14	12 (0.4)	0.02 (0.01–0.03)	NA
15–24	94 (3.0)	0.13 (0.10–0.15)	NA
25–34	302 (9.8)	0.39 (0.34–0.43)	NA
35–44	368 (11.9)	0.45 (0.40–0.50)	NA
45–54	453 (14.7)	0.66 (0.60–0.73)	NA
55–64	490 (15.9)	1.02 (0.93–1.11)	NA
65–74	603 (19.5)	1.70 (1.56–1.84)	NA
75–84	553 (17.9)	2.43 (2.23–2.64)	NA
>85	206 (6.7)	2.56 (2.21–2.91)	NA

*NA, not applicable. †Crude rates are used for the age category.

Most decedents whose death was associated with coccidioidomycosis were white (1,693 [54.8%]), 747 (24.2%) were Hispanic, 392 (12.7%) were black, 178 (5.8%) were Asian, and 79 (2.6%) were Native American (Table 2) Age-adjusted mortality rate was highest among Native Americans (2.56 deaths/1 million person-years), followed by Hispanics (1.77 deaths/1 million person-years) and lowest among whites (0.40 deaths/1 million person-years). Age-adjusted race-specific rates were elevated for all nonwhite groups. The likelihood of dying with coccidioidomycosis listed on the death record was 6.34× (95% CI 6.04–6.65) greater for Native Americans, 4.38× (95% CI 4.17–4.60) greater for Hispanics, 2.82× (95% CI 2.69–2.97) greater for Asians, and 1.70× (95% CI 1.61–1.80) greater for blacks than for whites.

**Table 2:** Concurrent conditions listed with coccidioidomycosis on death records, United States, 1990–2008*

Disease	ICD 9 code	ICD 10 code	No. deaths	Matched odds ratio
Vasculitis	446, 447.6	I776, I80, L95, M39, M31	36	6.55 (3.85–11.12)
Rheumatoid arthritis	714	M05-M06	45	6.51 (4.05–10.45)
Systemic lupus erythematosus	710	M32.0-M32.9	31	4.17(2.52–6.90)
HIV infection	042–0449	B20-B24	253	3.92 (3.24–4.75)
Tuberculosis	010.0–018.9	A16-A19	24	2.82 (1.66–4.79)
Diabetes mellitus	250.0–250.9	E11-E14	384	2.12 (1.86–2.42)
Chronic obstructive pulmonary disease	490–492, 494–496	J40-J44, J47	287	1.45(1.25–1.68)
Non-Hodgkin lymphoma	202, 200	C82-C85	47	1.44(1.03–2.01)

*ICD, International Classification of Diseases.

### Geographic Associations

All states reported coccidioidomycosis-associated deaths; however, most deaths occurred in California (1,451 [47.0%]) and Arizona (1,010 [32.7%]); age-adjusted mortality rates were 2.47 (95% CI 2.35–2.60) and 10.60 (95% CI 9.94–11.25) deaths per 1 million person-years, respectively (Figure 2). No notable temporal or seasonal trends were observed.

**Figure 2:** Number of coccidioidomycosis-associated deaths and age-adjusted rates per 1 million persons in the United States, 1990–2008.

### Disease Associations

Several conditions were more commonly represented on the death records of those whose death was associated with coccidioidomycosis than on the death records of matched controls whose death was not associated (Table 2.) These conditions are vasculitis (matched odds ratio [MOR] 6.55, 95% CI 3.85–11.12), rheumatoid arthritis (MOR 6.51, 95% CI 4.05–10.45), systemic lupus erythematosus (MOR 4.17, 95% CI 2.52–6.90), HIV infection (MOR 3.92, 95% CI 3.24–4.75), tuberculosis (MOR 2.82, 95%CI 1.66–4.79), diabetes mellitus (MOR 2.12, 95% CI 1.86–2.42), chronic obstructive pulmonary disease (MOR 1.45, 95% CI 1.25–1.68), and non-Hodgkin lymphoma (MOR 1.44, 95% CI 1.03–2.01).

## Discussion

The number of coccidioidomycosis-associated deaths in the United States is appreciable. Mortality rates were highest in persons >65 years of age, men, Native Americans, and Hispanics. Since 1997, however, coccidioidomycosis-related mortality rates have been relatively stable.

The increased risk for coccidioidomycosis-associated death among older persons might reflect decreasing immune function and increased prevalence of concurrent diseases. Increasing age has been identified as a potential risk factor for infection with *Coccidioides* spp (*28*). The increased rate of coccidioidomycosis-associated deaths observed among men might reflect their higher risk for severe pulmonary and disseminated coccidioidomycosis (*17*). The occupations associated with coccidioidomycosis (agricultural work, construction work, military service, and work at archeological sites) might also play an additional role (*10*–*12*) in the high numbers of coccidioidomycosis-associated deaths among men.

Age-adjusted, race-specific, coccidioidomycosis-associated mortality rates were highest for Native Americans and Hispanics; these rates probably reflect the higher density of American Indian and Hispanic populations living in areas that are arid and where coccidioidomycosis is endemic. All the coccidioidomycosis-associated deaths of Native Americans occurred in the western region of the United States. Some literature sources have suggested that Native Americans are at increased risk for exposure to *Coccidioides* spp. because of cultural practices and exposure to contaminated dust (*11*). Poor access to health care services might delay diagnosis, resulting in more severe disease. The high rates observed among Native Americans must be interpreted with caution, given the relatively small number of deaths.

Coccidioidomycosis-associated mortality rates were also higher among blacks and Asians than among whites but lower than rates among Native Americans and Hispanics. Black race and Filipino ancestry are recognized risk factors for disseminated disease (*2*). We were unable to ascertain coccidioidomycosis-associated mortality rates for Filipino Americans. These higher mortality rates might reflect an increased risk for severe disease, greater risk for exposure, or both.

That coccidioidomycosis-associated mortality rates are highest in Arizona and California is expected, given that *Coccidioides* spp. are endemic to these regions. These 2 regions are also classic retirement magnets; they attract elderly persons to migrate and settle down (*29*), thereby introducing new, unexposed populations to *Coccidioides* spp. Every state recorded coccidioidomycosis-associated deaths, which probably reflects population mobility and movement in and out of coccidioidomycosis-endemic areas after exposure.

Chronic illnesses have changed the way opportunistic mycoses affect the population. The conditions that were associated with coccidioidomycosis were all inherently associated with immunosuppression: HIV, tuberculosis, diabetes mellitus, autoimmune diseases, organ transplant, and cancers of lymphatic cells (*30*–*38*). Despite relatively low numbers of cases, an association was found between coccidioidomycosis-associated deaths and lupus erythematosus, vasculitis, and rheumatoid arthritis.

Several limitations are inherent when multiple-cause-of-death data are used. Although these data are population based and contain large numbers of observations, death certificates probably underreport causes of death and can contain errors, which have been attributed to a variety of factors (*39*). Mortality rates can be distorted because of errors in population estimates, particularly for race/ethnicity. Because estimates of the at-risk population factor into the denominator for rate calculations, such errors can lead to biased estimates. Although inferential statistics are not designed for use with population-based data, 95% CIs demonstrate that error does exist in the mortality rates and rate ratios reported here. We urge caution in the strict interpretation of our values.

Coccidioidomycosis remains a major cause of death in the United States. Given the growing US population of elderly and immunosuppressed persons, the number of coccidioidomycosis-related deaths will probably increase, resulting in higher costs to the health care system (*38*). Effects of increasing health care costs associated with coccidioidomycosis have been observed in coccidioidomycosis-endemic states; almost half of the reported case-patients are hospitalized and make multiple visits to emergency rooms and outpatient facilities during the course of the illness (*15*). Physicians should be aware of the increased risk for coccidioidomycosis-associated death among those who are immunosuppressed, elderly, male, Hispanic, and/or Native American. For identifying suspected cases, an accurate travel exposure and occupational history are crucial, especially in persons from non–coccidioidomycosis-endemic areas. Further investigation into measures that will effectively decrease coccidioidomycosis exposure risk to the general public is needed, as are more studies of health disparities that surround coccidioidomycosis-associated deaths.

## References

[1] Fisher MC, Koenig GL, White TJ, Taylor JT. Molecular and phenotypic description of *Coccidioides posadasii* sp nov, previously recognized as the non-California population of *Coccidioides immitis.* Mycologia. 2002;94:73–84. 10.2307/376184721156479

[2] Kirkland TN, Fierer J. Coccidioidomycosis: a reemerging infectious disease. Emerg Infect Dis. 1996;2:192–9. 10.3201/eid0203.9603058903229PMC2626789

[3] Hector RF, Rutherford GW, Tsang CA, Erhart LM, McCotter O, Anderson SM. The public health impact of coccidioidomycosis in Arizona and California. Int J Environ Res Public Health. 2011;8:1150–73. 10.3390/ijerph804115021695034PMC3118883

[4] Flaherman VJ, Hector R, Rutherford GW. Estimating severe coccidioidomycosis in California. Emerg Infect Dis. 2007;13:1087–90. 10.3201/eid1307.06148018214188PMC2878228

[5] Galgiani JN, Neil AM, Blair JE, Catanzaro A, Johnson RH, Stevens DA, Coccidioidomycosis. Clin Infect Dis. 2005;41:1217–23. 10.1086/49699116206093

[6] California Department of Public Health. Coccidioidomycosis yearly summary report 2001–2010 [cited 2012 Jul 9]. http://www.cdph.ca.gov/data/statistics/Documents/COCCIDIOIDMYCOSIS.pdf

[7] Arizona Department of Health Services. Coccidioidomycosis surveillance data annual report 2010 [cited 2012 Jul 9]. http://www.azdhs.gov/phs/oids/epi/disease/cocci/surv_dat.html

[8] US Census Bureau. State & county quickfacts [cited 2012 Jul 9]. http://quickfacts.census.gov/qfd/states/04000.html

[9] Drutz DJ, Huppert M. Coccidioidomycosis: factors affecting the host–parasite interaction. J Infect Dis. 1983;147:372–90. 10.1093/infdis/147.3.3726300253

[10] Pappagianis D. Marked increase in cases of coccidioidomycosis in California: 1991, 1992, and 1993. Clin Infect Dis. 1994;19:S14–8. 10.1093/clinids/19.Supplement_1.147948566

[11] Petersen LR. Coccidioidomycosis among workers at an archeological site, northeastern Utah. Emerg Infect Dis. 2004;10:637–42. 10.3201/eid1004.03044615200853PMC3323065

[12] Cummings KC. Point-source outbreak of coccidioidomycosis in construction workers. Epidemiol Infect. 2010;138:507–11. 10.1017/S095026880999099919845993

[13] Pappagianis D; Coccidioidomycosis Serology Laboratory. Coccidioidomycosis in California state correctional institutions. Ann N Y Acad Sci. 2007;1111:103–11. Epub 2007 Mar 1. 10.1196/annals.1406.01117332089

[14] Centers for Disease Control and Prevention. Case definitions for infectious conditions under public health surveillance, coccidioidomycosis [cited 2012 Jun 28]. http://www.cdc.gov/osels/ph_surveillance/nndss/casedef/introduction.htm

[15] Tsang CA, Anderson SM, Imholte SB, Erhart LM, Chen S, Park BJ. Enhanced surveillance of coccidioidomycosis, Arizona, USA, 2007–2008. Emerg Infect Dis. 2010;16:1738–44. 10.3201/eid1611.10047521029532PMC3294516

[16] Johnson RH, Einstein H. Coccidioidal meningitis. Clin Infect Dis. 2006;42:103–7. 10.1086/49759616323099

[17] Rosenstein NE, Emery KW, Werner SB, Kao A, Johnson R, Roger D. Risk factors for severe pulmonary and disseminated coccidioidomycosis: Kern County, California, 1995–1996. Clin Infect Dis. 2001;32:708–14. 10.1086/31920311229838

[18] Spinello IM, Munoz A, Johnson R. Pulmonary coccidioidomycosis. Semin Respir Crit Care Med. 2008;29:166–73. 10.1055/s-2008-106385518365998

[19] Pfaller MA, Diekema D. Epidemiology of invasive mycoses in North America. Crit Rev Microbiol. 2010;36:1–53. 10.3109/1040841090324144420088682

[20] McNeil MM, Nash S, Haijeh R. Trends in mortality due to invasive mycotic disease in the United States, 1980–1997. Clin Infect Dis. 2001;33:641–7. 10.1086/32260611486286

[21] Centers for Disease Control and Prevention. Bridged-race intercensal estimates of the July 1, 1990–July 1, 1999, United States resident population by county, single-year of age, sex, race, and Hispanic origin, prepared by the US Census Bureau with support from the National Cancer Institute [cited 2010 Jan 12]. http://www.cdc.gov/nchs/about/major/dvs/popbridge/popbridge.htm

[22] Centers for Disease Control and Prevention. Estimates of the July 1, 2000–July 1, 2006, United States resident population from the vintage 2006 postcensal series by year, county, age, sex, race, and Hispanic origin, prepared under a collaborative arrangement with the US Census Bureau [cited 2010 Jan 12]. http://www.cdc.gov/nchs/about/major/dvs/popbridge/popbridge.htm

[23] World Health Organization. International Classification of Diseases, 9th revision. Geneva: The Organization; 1980.

[24] World Health Organization. International Classification of Diseases, 10th revision. Geneva: The Organization; 1992.

[25] Anderson RN, Rosenberg HM. Age standardization of death rates: implementation of the year 2000 standard. National vital statistics reports, vol. 47, no. 3. Hyattsville (MD): National Center for Health Statistics; 1998.9796247

[26] Gardner JW, Sanborn JS. Years of potential life lost (YPLL)—what does it measure? Epidemiology. 1990;1:322–9. 10.1097/00001648-199007000-000122083312

[27] Centers for Disease Control and Prevention. Web-based Injury Statistics Query and Reporting System [cited 2010 Jan 15]. http://www.cdc.gov/injury/wisqars/index.html

[28] Blair JE, Mayer AP, Currier J, Files JA, Wu Q. Coccidioidomycosis in elderly persons. Clin Infect Dis. 2008;47:1513–8. 10.1086/59319218990061

[29] Frey WH, Liaw K, Lin G. State magnets for different elderly migrant types in the United States. Int J Popul Geogr. 2000;6:21–44. 10.1002/(SICI)1099-1220(200001/02)6:1<21::AID-IJPG157>3.0.CO;2-S

[30] Burwell LA, Park BJ, Wannemuehler KA, Kendig N, Pelton J, Chaput E, Outcomes among inmates treated for coccidioidomycosis at a correctional institution during a community outbreak, Kern County, California, 2004. Clin Infect Dis. 2009;49:e113–9. 10.1086/64811919886797

[31] Rosenstein NE, Emery KW, Werner SB, Kao A, Johnson R, Rogers D, Risk factors for severe pulmonary and disseminated coccidioidomycosis: Kern County, California, 1995–1996. Clin Infect Dis. 2001;32:708–15. 10.1086/31920311229838

[32] Centers for Disease Control and Prevention. Increase in coccidioidomycosis—California, 2000–2007. MMWR Morb Mortal Wkly Rep. 2009;58:105–9.19214158

[33] Selik RM, Karon JM, Ward JW. Effect of the human immunodeficiency virus epidemic on mortality from opportunistic infections in the United States in 1993. J Infect Dis. 1997;176:632–6. 10.1086/5140839291308

[34] Singh VR, Smith DK, Lawerence J, Kelly PC, Thomas AR, Spitz B, Coccidioidomycosis in patients infected with human immunodeficiency virus: review of 91 cases at a single institution. Clin Infect Dis. 1996;23:563–8. 10.1093/clinids/23.3.5638879781

[35] Cadena J, Hartzler A, Hsue G, Longfield RN. Coccidioidomycosis and tuberculosis coinfection at a tuberculosis hospital: clinical features and literature review. Medicine (Baltimore). 2009;88:66–76. 10.1097/MD.0b013e318194757a19352301

[36] Bergstrom L, Yocum DE, Ampel NM, Villanueva I, Lisse J, Gluck O, Increased risk of coccidioidomycosis in patients treated with tumor necrosis factor alpha antagonists. Arthritis Rheum. 2004;50:1959–66. 10.1002/art.2045415188373

[37] Santelli AC, Blair JE, Roust LR. Coccidioidomycosis in patients with diabetes mellitus. Am J Med. 2006;119:964–9. 10.1016/j.amjmed.2006.03.03317071165

[38] Park BJ, Sigel K, Vaz V, Komatsu K, McRill C, Phelan M, An epidemic of coccidioidomycosis in Arizona associated with climatic changes, 1998–2001. J Infect Dis. 2005;191:1981–7. 10.1086/43009215871133

[39] Pritt BS, Hardin NJ, Richmond JA, Shapiro SL. Death certification errors at an academic institution. Arch Pathol Lab Med. 2005;129:1476–9.1625303010.5858/2005-129-1476-DCEAAA

# Research article: Coccidioidomycosis-associated Deaths, United States, 1990–2008

**DOI:** 10.3201/eid1811.120752

**Authors:** Jennifer Y. Huang, Benjamin Bristow, Shira Shafir, Frank Sorvillo

**Affiliations:** Keck School of Medicine of University of Southern California, Los Angeles, California, USA (J.Y. Huang);; and University of California Los Angeles Fielding School of Public Health, Los Angeles (B. Bristow, S. Shafir, F. Sorvillo)

## Medscape CME Quiz

## Earning CME Credit

To obtain credit, you should first read the journal article. After reading the article, you should be able to answer the following, related, multiple-choice questions. To complete the questions (with a minimum 70% passing score) and earn continuing medical education (CME) credit, please go to www.medscape.org/journal/eid. Credit cannot be obtained for tests completed on paper, although you may use the worksheet below to keep a record of your answers. You must be a registered user on Medscape.org. If you are not registered on Medscape.org, please click on the New Users: Free Registration link on the left hand side of the website to register. Only one answer is correct for each question. Once you successfully answer all post-test questions you will be able to view and/or print your certificate. For questions regarding the content of this activity, contact the accredited provider, CME@medscape.net. For technical assistance, contact CME@webmd.net. American Medical Association’s Physician’s Recognition Award (AMA PRA) credits are accepted in the US as evidence of participation in CME activities. For further information on this award, please refer to http://www.ama-assn.org/ama/pub/category/2922.html. The AMA has determined that physicians not licensed in the US who participate in this CME activity are eligible for *AMA PRA Category 1 Credits*™. Through agreements that the AMA has made with agencies in some countries, AMA PRA credit may be acceptable as evidence of participation in CME activities. If you are not licensed in the US, please complete the questions online, print the certificate and present it to your national medical association for review.

## Article Title:

## Coccidioidomycosis-associated Deaths, United States, 1990–2008

## CME Questions

**1. You are seeing a 45-year-old man with a 4-week history of cough and fever. He was visiting a cousin in the southwestern United States last month, and you consider whether he might have coccidioidomycosis. What should you consider regarding the epidemiology of coccidioidomycosis?**

A. *Coccidioides* spp. are usually found at high elevations

B. *Coccidioides* spp. are usually found in wet climates

C. Up to 50% of people in endemic areas have been exposed to *Coccidioides* spores

D. *Coccidioides* spp. are found on the surface of the soil

**2. What should you consider regarding the transmission and clinical course of coccidioidomycosis?**

A. Livestock frequently transmit coccidioidomycosis to humans

B. Person-to-person transmission accounts for most cases of coccidioidomycosis

C. One quarter of patients with coccidioidomycosis develop extrapulmonary disease

D. The rate of disseminated disease is higher among men vs women

**3. The patient is diagnosed with coccidioidomycosis. What should you consider regarding the demographics of fatal cases of coccidioidomycosis in the current study?**

A. There was no sex-based difference in the risk of mortality

B. The highest rate of death was among children and adolescents

C. Race and ethnicity did not affect the risk of mortality

D. Native Americans experienced the highest rate of mortality compared with other racial/ethnic groups

**4. The patient has an extensive past medical history. Which of the following comorbid illnesses was most associated with a higher risk of death among patients with coccidioidomycosis in the current study?**

A. Rheumatoid arthritis

B. Congestive heart failure

C. Chronic obstructive pulmonary disease

D. Recurrent dental abscesses

## Activity Evaluation

**Table:** 

**1. The activity supported the learning objectives.**				
Strongly Disagree				Strongly Agree
1	2	3	4	5
**2. The material was organized clearly for learning to occur.**				
Strongly Disagree				Strongly Agree
1	2	3	4	5
**3. The content learned from this activity will impact my practice.**				
Strongly Disagree				Strongly Agree
1	2	3	4	5
**4. The activity was presented objectively and free of commercial bias.**				
Strongly Disagree				Strongly Agree
1	2	3	4	5
